# Letters from Paris

**Published:** 1849-10-01

**Authors:** 


### CORRESPONDENCE FROM PARIS.

Paris is in some measure recovered from the state of desolation which civil war and discord had produced, and once again science rears her holy head. Many of the physicians who had deserted the treatment of the maladies of the human frame for those which affect "the state politic" have found that they had better return to their old pursuits, as well for the benefit of the public as of themselves. Trelat, Recurt, and other men of eminence in the profession, have very wisely resumed their practice, and left the fitful fevers of popular commotions for the calmer, and, after all, the more ennobling duties of the physician. And doubtless poor Raspail, now shut up in prison, for taking a part in the demonstration of June last, which might have led to his being a member of the Provisional Government, would prefer prescribing his favourite camphor in every case that presented itself. This worthy person is certainly gifted with considerable talent, but is remarkable for the adhesive determination with which he fixes on one idea, whether in politics or in medical science; and in the latter department he has written much, and has even written well; but all his thoughts seem concentrated upon his favourite drug. He has pointed out one of the powers of camphor, which, in a psychological point of view, is most important—that of putting a stop to that fearful insomnolence which accompanies the incubation and first development of insanity; when opium, hyoscyamus, conium, stramonium, and "all the drowsy syrups of the east" fail to produce any effect, a grain of camphor, formed into a pill, followed by a draught of an ounce and a half of the infusion of hops, with five drops of sulphuric ether, is his usual remedy for procuring sleep, and a more delicious balm to the excited mind does not exist. A gentle slumber, even in the most excited cases, generally follows, and the patient awakes refreshed, calm, and composed. His cigarettes of camphor are in much repute amongst the labouring classes here, and seem to produce a state somewhat analogous to opium smoking; the reveries which follow upon their use have, however, less of the charm of exciting the imagination.

The Academy of Science has been actively employed in its various departments, and greater energy in the consideration of the different subjects has been evinced. There is at present a very important point occupying the attention of the members, and also those of many of the members of the different scientific continental societies; but which, at present, remains a matter of controversy to be decided when more accurate experiments can be performed. It is asserted, and also shown by trials of different individuals, that the mind can so act upon the nervous system that it can create an electro-galvanic current, and direct it through the human body, so as to cause the needle of the compass to deviate from its usual position. That this marvellous power exists is denied by several of those who have been engaged experimentally in the controversy, and they affirm that the galvanometer is an instrument of such excessive delicacy that it does not afford true indications; that it is acted upon by such minute and almost inappreciable circumstances, that it cannot be depended upon on occasions of extreme nicety of observation. The curious phenomena which are developed require much further investigation, and we shall be enabled hereafter to judge of the correctness of the doctrines which have been promulgated by the original observers.

Dr. Cheneau has presented a memoir to the Academy on the treatment of epilepsy which is well worthy perusal. He desires to prove that the disease is curable by medicine, resting his principal mode of arresting its progress upon the judicious employment of digitalis. The cases that have proved the most inveterate have yielded to a perseverance in the use of this remedy for a period of six or eight months. He has submitted six instances to the Academy, in which he has been successful; the first occurred at the Bicêtre, under the care of Dr. Voisin. A young man, aged twenty, had been epileptic, it is supposed from fright, from his fourteenth year; two months' treatment restored him to health. The second was one in which the disease, produced at the age of thirteen by fright, had lasted till the individual had attained his forty-second year. The treatment commenced the 20th of April, 1846, and after the 10th of June in the same year he had no attacks, which before had appeared, it is true, at only very long intervals; as many as six years having at one time elapsed without any access of paroxysm. The third case is certainly one as singular as any that has been registered upon the rolls of medical science. A young lady, thirteen years and a half old, had been subject for several years to the disease which had at length brought on idiocy and paralysis of half the body; the paroxysms were not very frequent, but were of great violence frightening the persons who nursed her, the countenance wearing a purple and almost a black hue, which sometimes lasted twelve hours after the fit had ceased; the hermiplegia, which was of the right side, prevented the movement of the limbs, and partially affected sensation. The treatment was commenced on the 4th of July, 1846, and by the month of January in the following year, the epileptic fits altogether ceased. A year has elapsed since the young lady has been able to go on with her education, and she is also able to run in the garden and to amuse herself with gymnastic exercises. The fourth notice is that of a young girl of ten years of age, upon whom epilepsy supervened after fright; it had lasted two years, but soon yielded to the usual remedy of Dr. Cheneau. The fifth case was that of a patient in the Bicêtre; he was, when placed under the treatment, sixteen years of age, and had been afflicted by the malady since he was five years old. At first, the paroxysms were but slight; he suddenly turned himself mechanically round any object immediately in his neighbourhood; this lasted about a minute. As the disease advanced, he had convulsions, and the fits became very frequent. The remedies were commenced on the 2nd of October, 1847, and in January, in the following year, was his last attack. Since that time his health has become perfectly established. The last case carries with it the same interest as the preceding ones. A boy, of ten years of age, after having been for three years subject to frequent fits, once as many as fourteen in twenty-four hours, was subjected to the use of the digitalis, and at the end of two months was an instance of the excellence of the system pursued by Dr. Cheneau. The essay is well written, and certainly demonstrates that digitalis, properly combined, has cured epilepsy, even when complicated with paralysis and with idiotism; that the cure has not been confined to youth, but has been decided even at an advanced age, and that the time occupied has not been of great duration. An incidental subject of discussion has been the subject of the sudden paleness of the face which has been noted down by the nosologists as one of the symptoms of epilepsy. One of the characteristics, as given by Georget, in the "Dictionnaire de Medicine," is "extreme pallor of the face, suddenly coming on towards the end of the fit, succeeding to a redness more or less intense which previously existed." In the hospital of the Bicêtre, one hundred and twenty fits have been watched with the most scrutinizing care, and this state has never once presented itself; on the contrary, the redness came on during the paroxysm, continued throughout the convulsions, and often lasted for some time afterwards. On no one occasion was the sudden paleness observed.

The rotatory motion of the head, upon which M. Belhomme wrote in the year, 1839, which is observable in sheep, the ox, the horse, and the dog, and which has been noticed in man, evidently a form of epilepsy, was considered by him to be always dependent upon the presence of hydatids in the cerebral substance. This opinion is now combated, and apparently with sufficient reason, for it was thought to be incurable, or that one treatment could alone give the slightest prospect of success—that of trepanning the cranium, and the removal of the hydatids, or whatever foreign substance might be present. M. Leblanc, one of the most eminent veterinary surgeons of France, has frequently cured the disease in horses by the affusion of cold water upon the head. In such cases, it is not unlikely that the increased mucous discharge that follows, may carry with it entozoa, which may have produced a degree of irritation upon the nervous system sufficient to cause the rotatory convulsions, which seem to be a spasmodic contraction of the muscles, something similar to those which present themselves under other circumstances.

It is not unusual to observe as one of the precursory symptoms of epilepsy, an extraordinary impetus in the invalid, which directs him, in spite of every obstacle, to rush forward in a straight line, and if anything arrests his progress, he resumes it as quickly as possible, or rushes forward in another direction. M. Vallée, who has under his charge the idiots of the Bicêtre, has remarked the case of an idiot who was suddenly seized with the irresitible desire of motion, rushed forward, and when he found the wall imposing its mechanical resistance, he pushed with his head and his abdomen against it, and continued to make all the movements of running against it until vertigo came on. If stopped by the persons whose care it was to watch over him, he often contrived to escape, and would commence again precisely as he had before done, and would go on until the crisis came on, which compelled him to discontinue this involuntary muscular movement. The French psychologists give the name of tournis to the rotatory motion, both in men and in animals, and we shall, doubtless, have further illustrations of this singular malady, besides those furnished to us by the veterinary schools.

The number of patients in the different private lunatic asylums which, under the name of Maisons de Santé, exist in different parts of Paris, has been considerably increased within the last two years, owing to the alterations produced in fortune by the vicissitudes of the revolution, as well as to the excitement which has been caused in the minds of men by the singular events that have occurred, it is understood that Brierre de Boismont, who has in the Rue Neuve Sainte Geneviève an establishment of considerable notoriety, is about to publish some observations made by him during that period. The House of Ivry, under the direction of Baillarger, Moreau, and Mitivre, and which was originally built by Esquirol as the model of a mansion intended for the reception of lunatics, has been unusually crowded with persons upon whom political matters have produced an influence. Madame de Saint Marcel has for some time been considered as having set the example of giving peculiar facilities for individual practitioners to place their patients where they can be under their own management, instead of the head of the establishment, who is usually devoted to the peculiar class of maladies, whether this is an improvement remains yet to be proved.

Dr. Junod is anxious to proceed to England to introduce into practice his invention of the exhausted air-boot for diseases of the brain. He places a large metallic tube upon the leg, which is exactly in the shape of a boot, and by an apparatus connected with it he gradually completely exhausts it of air, the consequence of which is the limb swells enormously, becoming three times the ordinary size, there is an immense quantity of blood thus determined to the lower extremity, whose capillary vessels become much distended, and relief is thus given to the overloaded sanguineous system elsewhere, which is oftentimes permanent, as the limb only slowly unloads itself from the humors driven into it, there is no reaction to be feared, the objection that at first presents itself is the probability of its giving rise to varicose veins, but from the numerous experiments that have been tried, it would appear that this effect has not been produced. It would appear to offer a resource where there was great determination of arterial blood to the head, or where there is venous retardation. Several cases have been placed under the care of Dr. Junod by the faculty of Paris, with a view of giving him an opportunity of introducing this novel instrument into practice, but he has announced his intention of going to London as soon as the medical schools are opened there, that he may show of what he is capable.

There is, I am happy to learn, a prospect of the revival of the periodical devoted to psychology, which, under the immediate auspices of Baillarger, Cerise, and Longet, produced such valuable papers before the republic was declared, it is not unlikely that a new number will very soon make its appearance, there are so many trials occurring in different parts of France from time to time, which throw a fresh light upon the vagaries and inconsistencies of the human mind, which require to be recorded, that it is deeply to be regretted that the work which commenced with such favourable results should have been so prematurely drawn to a close.

Amongst the numerous residents of Paris is Mr. Dyce Sombre, whose case has been so lately the theme of legal discussion. He is considering the best means of bringing before the public a narrative of what he has had to encounter since his sanity has been called into question. An autobiography from his pen would doubtless be interesting. He has lately drawn up a codicil to his will, and invited three physicians to witness it; they, of course, complied with his wish, as they only testify to their having seen him perform the mechanical act of signing his name to certain documents, without giving the slightest opinion as to his mental capability of disposing of his property, or of his power of forming a correct judgment, it is considered by them as giving evidence that it is his own handwriting whilst in a perfect state of bodily health.

It is a curious fact, that long protracted cases of catalepsy are not so uncommon in France as they are in England. Scarcely had the marvellous case at Nantes, which has been the theme of so much conversation and discussion terminated, when a new one presented itself in the department of the Aube. A young female, not many months married, the wife of a small landed proprietor, has now been for eighteen days in that extraordinary state of trance which seems to defy all explanation. The sleep is tranquil, completely uninterrupted by movement of any description; the secretions are much diminished, and there have been no excretions; the breathing is easy, somewhat slower than under ordinary circumstances; the pulse is a mere thread. The marvel, as it may be supposed, excites the greatest curiosity, and individuals from all parts of the country, medical and non-medical, go to witness it.

At the last meeting of the Academy of Science, M. du Couxet, who has lately returned from a residence in Africa, read a paper interesting in an ethnological and psychological point of view. Some of the facts seem to border upon the extravagant; they are, however, borne out by the testimony of credible witnesses. In the kingdom of Gondar there is an original race of men who have a remarkable zoological peculiarity,—a candal appendix, formed by a prolongation of the vertebral column, which bears very much the semblance of a tail. Their mental character corresponds with the idea that has existed, that animals with this distinctive formation are scarcely to be ranked in the genus-homo. Their intellect seems to be of the lowest gradation; their physical form is hideous. There are some of these beings who have been taken as slaves; one was in the possession of the Emir of Mecca, and had some share of intelligence, spoke the Arabic language, and described the nation from amongst whom he came as living in the neighbourhood of Sennal, worshipping the sun, the moon, and some of the stars; they immolated victims, whom they ate at the shrine of the great serpent, without sparing either age or sex. It appeared that this individual had an irresistible propensity to eat raw flesh; it returned upon him periodically, and the greatest care was taken that the limb of an animal should be placed in his way at fixed times, which he devoured apparently in a paroxysm of rage. He spoke of the feeling which seized him with some expression of fear, lest he should be tempted in the absence of some other food to seize upon an infant and devour it. This race, much smaller than the negro, generally is not above five feet in height, and very much resembles the monkey tribe. They are badly proportioned; their arms are very long, and their feet and hands longer and flatter than amongst men generally. The forehead is very low, and runs backward; their ears are long and deformed; their eyes small, black, and brilliant; the nose large, but flat; the mouth large, with sharp white teeth; the lips thick; the hair curly, thin and short, but not woolly; the tail is about two or three inches long. A portrait, drawn from the individual who belonged to the Emir of Mecca, was placed before the inspection of the members of the academy.

Amongst the observations which have been made at different periods by medical men, is the predisposition which has been noticed amongst some of those actors upon the stage who have delighted us with their comic powers, to sink into a state of profound melancholy, without the possibility of any one assigning a reason for this phenomenon. In France, there have been several instances to which Pinel and Esquirol have referred; and the biography so well written by Mrs. Mathews of her husband, gives us some insight into his peculiar affliction. It now happens that the most distinguished comic actress of France, who has so often drawn down peals of irresistible laughter, and who is a great favourite of the English audience of the French theatre in London, suffers from a similar malady. She has been compelled to quit Paris, and to the great regret of every one, fears are entertained that, after fascinating the whole world, she will sink into a state of permanent incapability of exerting her talents. She has retired into the country under the immediate care of a highly scientific medical man, who has devoted his mind to psychological pursuits; but from all that is known of her state, doubts have arisen that she will ever be enabled to resume her career. The manifestations seem fortunately to be of the second class described by Esquirol, not those of much morbid sensibility, but rather those of depression. There is no exaggeration of imaginary evils, but an absence of even ordinary excitability. Absolute silence and reserve, without any dominant illusion, seem to be the general characteristic of this state of mind; it appears to have its periodical access and remission. In several instances, this apathetic state has been observed in individuals of natural comic talent and flow of spirit, to make its appearance immediately after a meal, as if it were influenced by digestion, and many have hence had recourse to fermented liquors, which, though for a time they exhilarate, leave their bad effects behind them. Many of our most delightful social companions have exhibited similar depression; amongst them we might class Dr. Maginn, Porson, and, in the latter part of his life, Sheridan.

---

